# A Comparison Study of Constitutive Equation, Neural Networks, and Support Vector Regression for Modeling Hot Deformation of 316L Stainless Steel

**DOI:** 10.3390/ma13173766

**Published:** 2020-08-26

**Authors:** Shin-Hyung Song

**Affiliations:** Department of Smart Automobile, Soonchunhyang University, 22 Soonchunhyang-ro, Sinchang-myeon, Asan-si, Chungcheongnam-do 31538, Korea; neuro2@sch.ac.kr

**Keywords:** 316L stainless, hot deformation, flow stress, neural network, support vector regression

## Abstract

In this research, hot deformation experiments of 316L stainless steel were carried out at a temperature range of 800–1000 °C and strain rate of 2 × 10^−3^–2 × 10^−1^. The flow stress behavior of 316L stainless steel was found to be highly dependent on the strain rate and temperature. After the experimental study, the flow stress was modeled using the Arrhenius-type constitutive equation, a neural network approach, and the support vector regression algorithm. The present research mainly focused on a comparative study of three algorithms for modeling the characteristics of hot deformation. The results indicated that the neural network approach and the support vector regression algorithm could be used to model the flow stress better than the approach of the Arrhenius-type equation. The modeling efficiency of the support vector regression algorithm was also found to be more efficient than the algorithm for neural networks.

## 1. Introduction

Stainless steel is an iron-based alloy that is widely used because it has greater wear and corrosion resistance than other alloys such as mild steels and low alloy steels [1]. Depending on the alloying contents used, stainless steel can have good strength and oxidation resistance in high temperature environments, as well as the desired properties for cryogenic environments and marine applications [1,2,3]. One of the main types of stainless steel is austenitic stainless steel, which contains Cr (12–25 wt.%), Ni (8–25 wt.%), and Mo (0–6 wt.%) and has good high temperature properties. Austenitic stainless steel has several different types and characteristics. For instance, stainless steel type 316 is a molybdenum-added alloy which has good resistance to pitting, high temperature creep, and high temperature oxidation [3]. Thus, this alloy is often applied in defence and nuclear applications [3]. Type 304 alloy contains about 18% Cr and about 8% Ni, and therefore is protected from the aggressive environment by passive films made of chromium oxides [4,5]. Therefore, because 304 stainless steel has good corrosion resistance, the alloy is widely used in the gas and the oil industries [4,5]. Meanwhile, due to its excellent oxidation and creep resistance in high temperature environments, stainless steel type 310S is used in nuclear power plants and chemical industries [6].

For the design and production of appropriate stainless-steel products, it is crucial that the deformation behaviors of the various types of stainless steel are thoroughly studied across a wide range of temperatures and strain rates, and a deformation model of the alloys should be developed. To date, many relevant studies have been conducted, including but not limited to a study on the deformation of 316 stainless steel under 0.1–100 s^−1^ of strain rate and 1173–1473 K temperature [7], a study on the compressive behavior of AISI 321 under the temperature range of 950–1100 °C and the strain rate of 0.01–1 s^−1^ [8], and a study on the compression deformation behavior of AISI 304 under the strain rate of 0.001–5 s^−1^ and the temperature of 800–1200 °C.

To date, a number of studies have attempted to model the flow behavior of austenite stainless steels through various deformation experiments [3,8,9,10,11,12]. For example, Haj et al. [8] carried out hot compression tests for 321 stainless steel at temperatures ranging from 950 to 1100 °C and strain rates ranging from 0.01 s^−1^ to 1 s^−1^. In that study, a constitutive equation based on the Zener–Hollomon parameter was used to model the relationship between temperature, strain rate, and flow stress. Nkhoma et al. [9] also employed a Zener–Hollomon parameter-based constitutive equation for modeling the hot deformation behavior of 321- and 304-type stainless steel under temperatures from 800 to 1200 °C and strain rates from 0.001 s^−1^ to 5 s^−1^. In another study, the impact behavior of austenite stainless steel was modeled by Lee et al. [10]. In that experiment, 316L-type stainless steel was deformed by compressive SHPB (split Hopkinson pressure bar) test at a strain rate of 0.001–7500 s^−1^, to study the flow behavior, and the flow stress was modeled using the KHL (Khan–Huang–Liang) model. Mandal et al. [11] performed high-temperature deformation tests for Alloy D9 under temperatures ranging from 1123 to 1523 K and strain rates ranging from 0.001 s^−1^ to 100 s^−1^. In that study, the flow stress of Alloy D9 was modeled using the Arrhenius-type equation. Gupta et al. [12] conducted hot tensile tests for 316-type stainless steel at 323–623 K temperatures and 0.0001–0.1 s^−1^ strain rates. In those tests, the flow stress of 316 stainless steel was modeled employing the Johnson–Cook model, a modified type of the Zerilli–Armstrong model, and the Arrhenius-type equation.

In addition to these traditional approaches, in a number of studies, the neural network model has also been employed to examine the hot deformation of alloys. For example, in the study by Gupta et al. [12], the neural network model was used to model hot deformation, and the results indicated that the neural network model had better performance than any of the other traditional constitutive models examined. Li et al. [13] conducted hot compression tests for Al–Zn–Mg alloy at temperatures from 340 to 500 °C and strain rates from 0.001 to 10 s^−1^. In that study, the flow stress of the Al–Zn–Mg alloy was modeled using the Arrhenius-type model and the neural network model. The results found that the neural network model was better than the Arrhenius-type model. In a different study, Sabokpa et al. [14] performed hot compression tests for Ti600 at 250–400 °C temperatures and 0.0001–0.01 s^−1^ strain rates, while using the Arrhenius-type equation and the neural network model to model the flow stress of Ti600. The results found that the neural network model had excellent performance.

A literature study indicates that approaches employing constitutive equations were successfully used in modeling the hot deformation of stainless alloy. In particular, the Arrhenius-type equation has been the most widely used. However, the effectiveness of the constitutive equations was often limited. Specifically, the complex nature of the hot deformation of metals makes it fundamentally difficult to estimate the flow behavior, and the nonlinearity of the relationship of deformation parameters with flow stress, as well as the dispersion which can be shown in the experimental data also causes difficulties in modeling the flow stress using constitutive equations [14]. It appears that the neural network approach should be used to complement the drawbacks of the constitutive equations described above. However, artificial neural network approaches have limited efficiency in model development and computation, since the accuracy and computation time of artificial neural networks are significantly affected by the modeling parameters, for example, the number of hidden layers and neurons. Therefore, there is a strong demand for adopting an algorithm that can be efficiently modeled and computed for predicting the flow stress of high temperature deformation of alloys.

In the present study, support vector machine-based regression was employed in modeling the flow stress of hot deformation. To this end, 316-type stainless steel was hot deformed at temperatures from 800 °C to 1000 °C and strain rates from 0.0002 s^−1^ to 0.02 s^−1^. The flow stress of the 316 stainless steel was modeled using the Arrhenius-type equation, a neural network model, and the support vector machine regression algorithm. The flow stresses were obtained by different models along with RMSE (root mean square error) values. A comparison of different models and a discussion were also provided.

## 2. Materials and Methods

**Figure 1:**
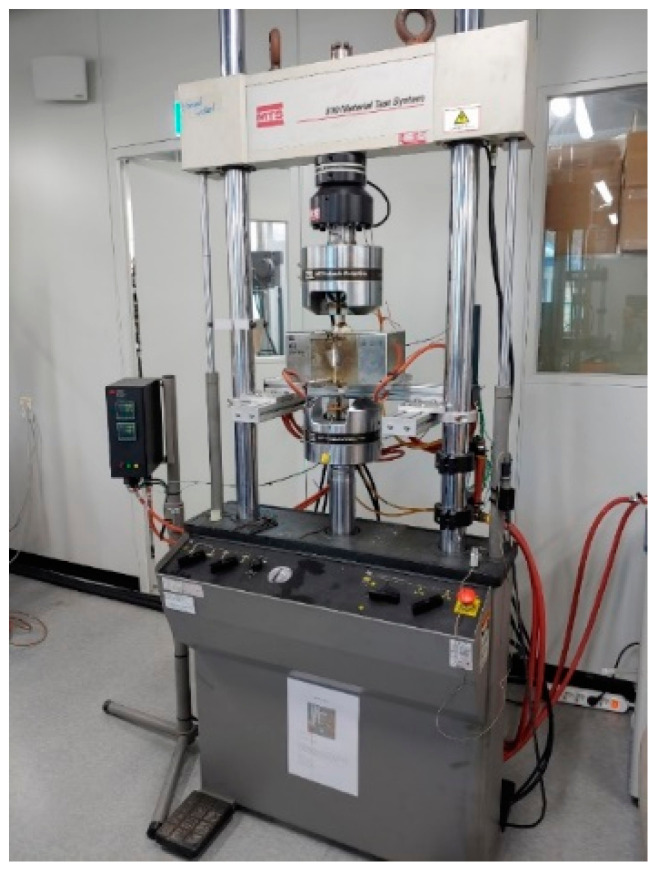
Equipment for tensile tests (MTS-810 material test system).

**Figure 2:**
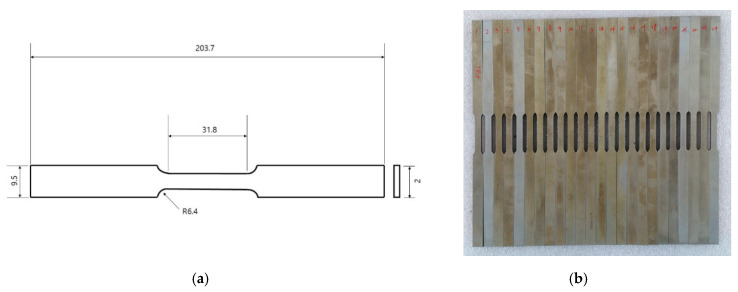
(**a**) Dimensions of test specimen(mm); (**b**) Prepared test specimens.

In the current study, hot tensile tests for 316-type stainless steel were done in the MTS-810 servo hydraulic material testing machine (Figure 1). The planar specimens for the tests were prepared by wire-EDM machining. Figure 2a shows the dimensions of the specimen and Figure 2b shows the actually prepared specimen. The high-temperature tensile experiments were done at strain rates of 0.0002 s^−1^, 0.002 s^−1^, and 0.02 s^−1^, at temperatures of 800 °C, 900 °C, and 1000 °C, for each strain rate.

**Figure 3:**
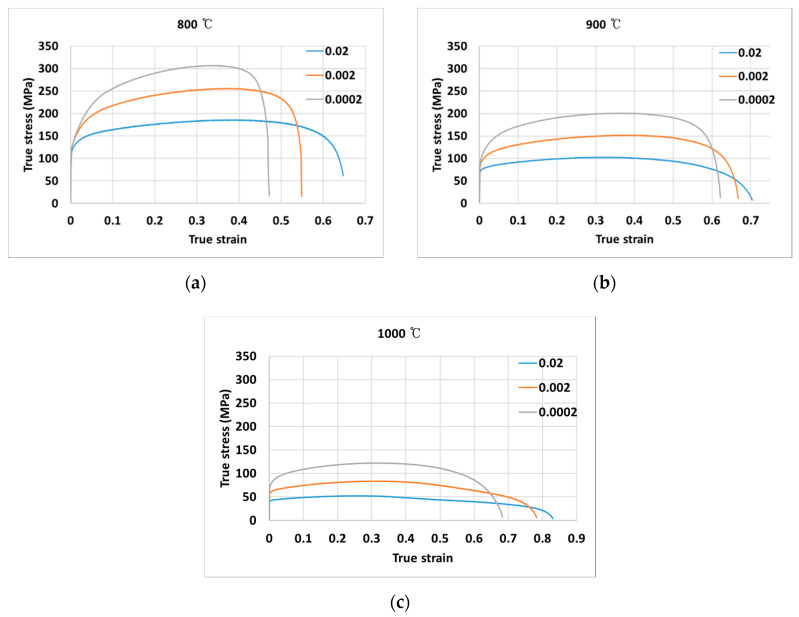
True stress-true strain curves at (**a**) 800 °C, (**b**) 900 °C, (**c**) 1000 °C.

The measured true stress-true strain curves for temperatures of 800 °C, 900 °C, and 1000 °C, respectively, are shown in Figure 3a–c, under varying strain rates of 0.0002 s^−1^, 0.002 s^−1^, and 0.02 s^−1^. Throughout the experiments, it is observed that flow stress is strongly dependent on the deformation strain rate and temperature. It is observed that as the temperature increases, the stress decreases; while as the stress increases, the strain rate increases.

### 2.1. Arrhenius Type Constitutive Modeling

In this study, the Arrhenius-type equation was employed to model the hot deformation of 316L stainless steel. The influences of temperature and strain rate on the flow behavior is represented by the Zener–Hollomon parameter, as shown below in Equation (1):

(1) $$Z\;=\;\dot{\varepsilon }\;\exp\left(\frac{Q}{RT}\right)$$

where

(2) $$\dot{\varepsilon }\;=\;AF\left(\sigma \right)\exp\left(\frac{-Q}{RT}\right)$$

(3) $$F\left(\sigma \right)\;=\;\{\begin{array}{c} \sigma^{n\prime }\;\alpha \sigma <0.8\\ \exp\left(\beta \sigma \right)\;\alpha \sigma >1.2\\ {\left[\sinh\left(\alpha \sigma \right)\right]}^{n}\;all\;\alpha \sigma \end{array}$$

Q represents the activation energy (J·mol^−1^); R represents the gas constant (8.314 J$\cdot {\mathrm{mol}}^{-1}K^{-1}$); T denotes the temperature (K); and $\dot{\varepsilon }$ is the strain rate ($s^{-1}$); while A, n′, α, β, and n represents material constants.

First, substituting the first Equation (3) into (2) and taking logarithms of both sides gives

(4) $$ln\sigma =\frac{1}{n^{\prime }}\;ln\dot{\varepsilon }-\frac{1}{n^{\prime }}lnB$$

**Figure 4:**
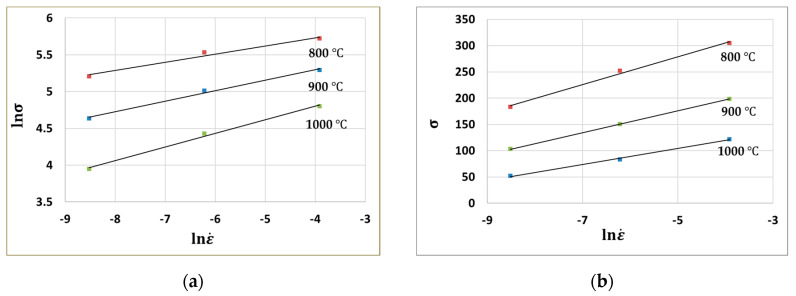
(**a**) ln$\sigma -\ln\dot{\varepsilon }$ plot and (**b**) $\sigma -\ln\dot{\varepsilon }$ plot when strain is 0.3.

Then, n′ can be obtained from the slopes of the ln$\sigma -\ln\dot{\varepsilon }$ curves. For instance, Figure 4a shows the plot of ln$\sigma -\ln\dot{\varepsilon }$ when the strain is 0.3.

Further, substituting the second Equation (3) into (2) and taking logarithms of both sides gives:

(5) $$\sigma \;=\;\frac{1}{\beta }ln\dot{\varepsilon }-\frac{1}{\beta }lnC$$

Similarly, β can be calculated from the slopes of the $\sigma -\ln\dot{\varepsilon }$ curves. Figure 4b shows the $\sigma -\ln\dot{\varepsilon }$ plot.

The material constant α is defined as follows:

(6) $$\alpha \;=\;\frac{\beta }{n^{\prime }}$$

Therefore, α is obtained after calculating β and $n^{\prime }$.

The material constant n can be obtained after substituting the last Equation (3) into (2) and taking logarithms of both sides. From the equation shown below, *n* can be calculated using the slopes of the $\ln\dot{\varepsilon }-\ln[\sinh\left(\alpha \sigma \right)]$ curves as:

(7) $$\ln[\sinh\left(\alpha \sigma \right)]\;=\;\frac{ln\dot{\varepsilon }}{n}+\frac{Q}{nRT}-\frac{lnA}{n}$$

**Figure 5:**
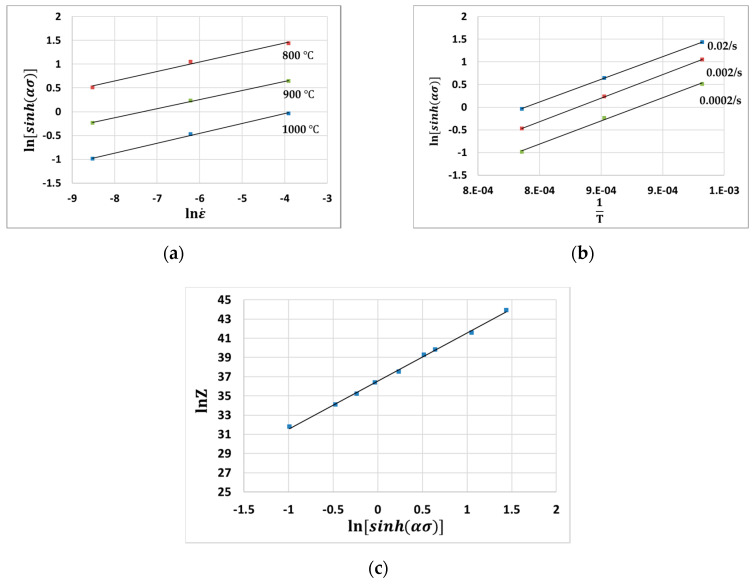
(**a**) ln$\sigma -\ln\dot{\varepsilon }$ plot and (**b**) $\ln[\sinh\left(\alpha \sigma \right)]-\left(\frac{1}{T}\right)$ (**c**) $\ln\dot{\varepsilon }-\ln[\sinh\left(\alpha \sigma \right)]$ plot when strain is 0.3.

Figure 5a shows the plot of $\ln\dot{\varepsilon }-\ln[\sinh\left(\alpha \sigma \right)]$ when the strain is 0.3. Similarly, the material constant Q can be calculated from the $\ln[\sinh\left(\alpha \sigma \right)]-\left(\frac{1}{T}\right)$ plot. Figure 5b shows the plot of $\ln[\sinh\left(\alpha \sigma \right)]-\left(\frac{1}{T}\right)$. Using Equations (1) and (7), one can obtain the following equation:

(8) lnZ = lnA+nln[sinh(ασ)]

From Equation (8), the material constant A can be calculated by using the y-intercept of the lnZ−ln[sinh(ασ)] plot, which is shown in Figure 5c.

After determining the material constants, one can obtain the equation describing the flow stress using Equations (1) and (2) in the form of the equation below. Table 1 shows the parameters obtained for the Arrhenius equation.

(9) $$\sigma \;=\;\frac{1}{\alpha }\ln[{\left(\frac{Z}{A}\right)}^{\frac{1}{n}}+{\left[{\left(\frac{Z}{A}\right)}^{\frac{2}{n}}+1\right]}^{\frac{1}{2}}]$$

### 2.2. Neural Networks

**Figure 6:**
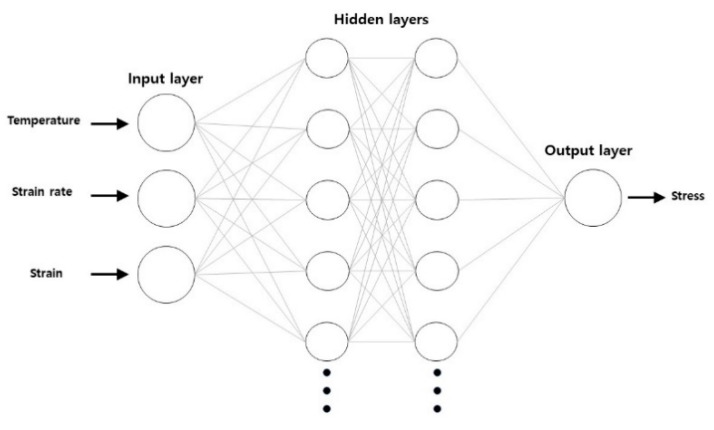
Neural network model.

Figure 6 is a schematic diagram of the developed neural networks. Each of the neural networks has three layers, namely input, hidden, and output layers. In this study, two hidden layers were used in the developed model. Each layer has neurons. For example, the input layer has three neurons, which represent the temperature, strain rate, and strain. Neurons are processed in terms of their weight numbers. During the operation of the neural networks, the numbers received by a neuron from the neurons in the previous layer are processed according to the weight number of the current neuron. After the calculation, the calculated number is delivered to the neurons in the next layer in order. In the current model, the final value is received by the output layer with one neuron, which represents the flow stress of hot deformation. The numbers of hidden layers and neurons in the hidden layers directly and significantly influence the accuracy of the prediction results. Gupta et al. [12] employed a single hidden layer with 15 hidden neurons. Li et al. [13] employed 18 hidden layers and Sabokpa et al. [14] employed 15 hidden neurons in a single hidden layer. In this research, the accuracy of the neural networks changed irregularly with the change of the numbers of hidden layers and neurons. Therefore, the ideal number of hidden layers and hidden neurons are decided after many simulations on a trial and error basis. In the current model, 250 neurons are used for each hidden layer. The activation function is the RELU (rectified linear unit) function. Training the neural network for good prediction accuracy involves obtaining the most appropriate set of weight values of neurons. For the current model, a back-propagation training algorithm with ADAM optimization was adopted. Mean square error is employed as a loss function for evaluating the performance of the neural networks during the training. Learning rate and weight decay are the parameters related to the amount being modified during the update of the weight of the neurons. In this research, 0.001 and 0.1 are chosen for learning rate and weight decay. In this research, the weight decay was set as 0.1 and the learning rate was set as 0.01. For the development of the neural networks, Keras (a Python package) was utilized. For the training data of the current model, 324 points of data were chosen randomly from nine stress-strain curves for the strain range from 0.05 to 0.45. Another 81 points of data from the same nine curves for the strain range from 0.05 to 0.45 were chosen to test the developed model. To train the neural networks, training data is normalized by the equation below:

(10) $$x^{i,\;nor}\;=\frac{x^{i}-x^{min}}{x^{max}-x^{min}}$$

Here, $x^{i,\;nor}$ is the normalized value of the ith index data; $x^{i}$ is the ith data; and $x^{max}$ and $x^{min}$ are maximum value and minimum value of data, respectively. Table 2 shows the process parameters for the developed neural network model.

### 2.3. SVR (Support Vector Regression)

**Figure 7:**
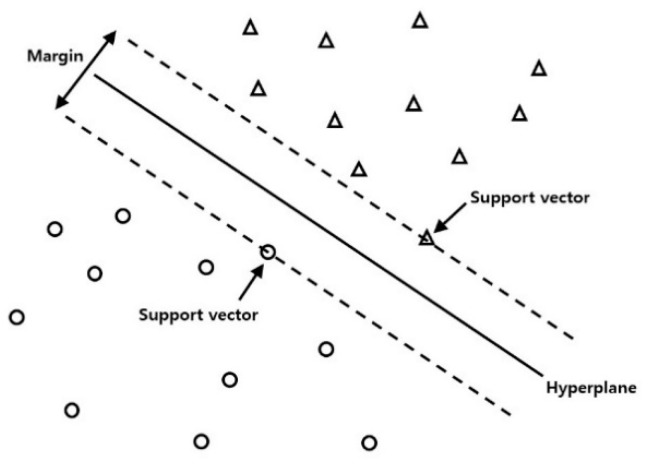
Support vector machine.

SVR (support vector regression) [15] is an applied type of SVM (support vector machine) classification algorithm. In SVR, the data is split into groups by hyperplane. The distance from the hyperplane to the data at each group’s boundary is called the margin, and the data on the marginal boundary is called the support vector. The purpose of training the SVR is to find the optimal hyperplane with the largest margin. Figure 7 shows a schematic diagram of the support vector machine algorithm with the original linear hyperplane. For the training of the current data, the relationship of input data with the output data is nonlinear and complex. Therefore, a new hyperplane is induced to a feature space by the kernel method to help train the nonlinear dataset. A Python package (Scikit-learn) was used in the current study. The regularization parameter is chosen to be 5000, and the epsilon value is chosen to be 0.003. Table 3 lists the process parameters for the developed SVR model.

## 3. Results and Discussion

### 3.1. Arrhenius-Type Constituitive Modeling

**Figure 8:**
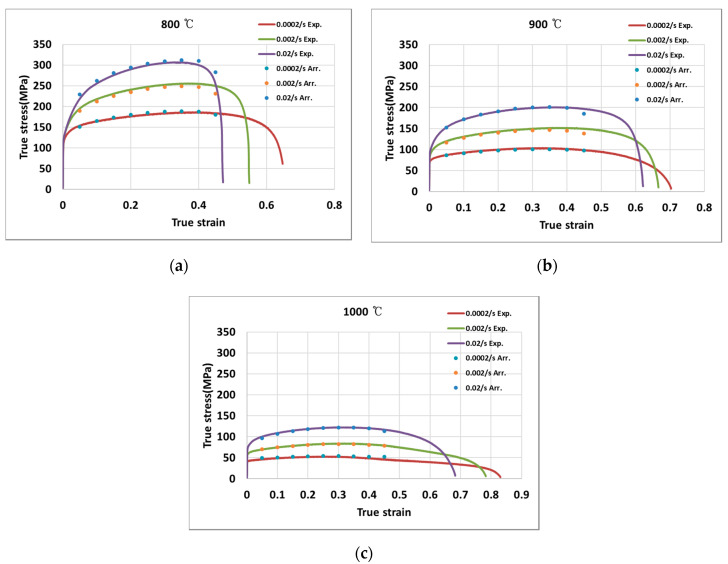
True stress-true strain curves at (**a**) 800 °C; (**b**) 900 °C; (**c**) 1000 °C.

Figure 8a–c shows the calculated true stress by the Arrhenius equation indicated on the experimental curves for 800 °C, 900 °C, and 1000 °C, respectively, when the strain rates are 0.0002 s^−1^, 0.002 s^−1^, and 0.02 s^−1^, respectively. The stresses predicted by the Arrhenius equation were close to the experimental values. The RMSE (root mean square error) for the stress by the Arrhenius equation was found to be 5.59.

### 3.2. Neural Networks

**Figure 9:**
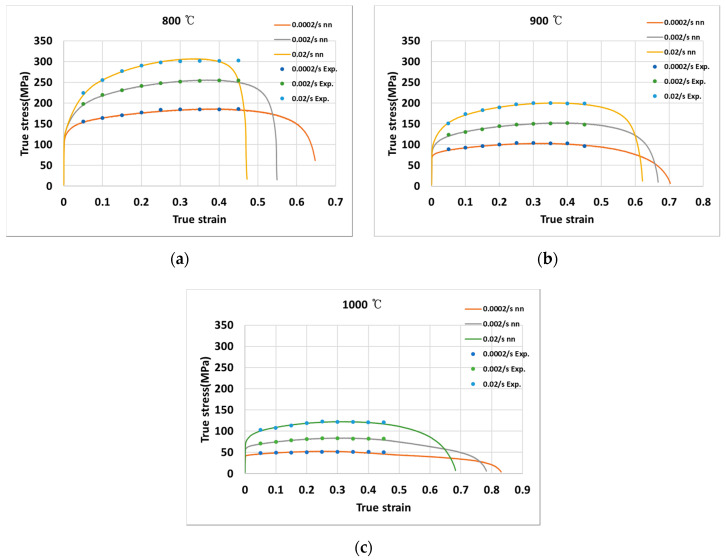
True stress-true strain curves at (**a**) 800 °C; (**b**) 900 °C; (**c**) 1000 °C.

Figure 9 shows the calculated true stress by the neural networks indicated on the measured stress for the temperatures of (a) 800 °C, (b) 900 °C, and (c) 1000 °C, when the strain rates are 0.0002 s^−1^, 0.002 s^−1^, and 0.02 s^−1^. The RMSE of the true stress using the neural network model was found to be 2.85. The results indicate that the true stress using the developed neural network is more similar to the measured values than the stress calculated by the Arrhenius equation. However, substantial effort was needed to obtain an appropriate model with good process parameters. In addition, the neural network model was computationally more expensive.

### 3.3. Support Vector Regression

**Figure 10:**
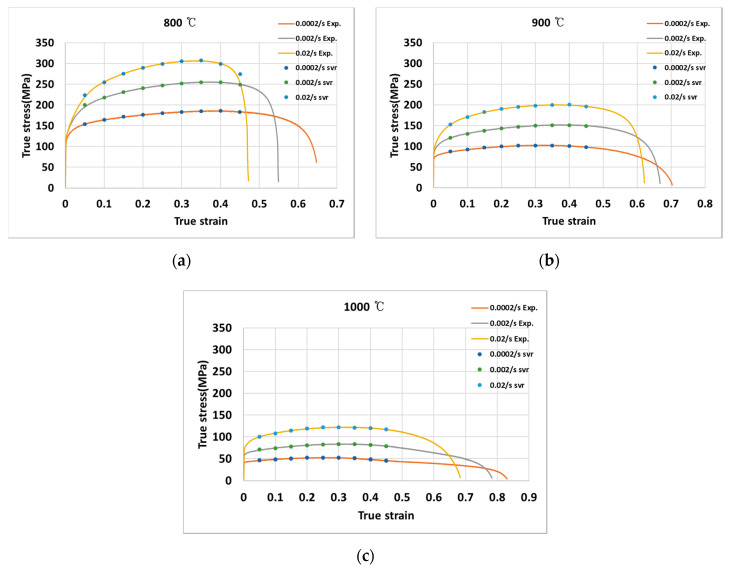
True stress-true strain curves at (**a**) 800 °C; (**b**) 900 °C; (**c**) 1000 °C.

Figure 10 shows the stress predicted using the support vector regression algorithm indicated with the measured stress at (a) 800 °C, (b) 900 °C, and (c) 1000 °C for the strain rates of 0.0002 s^−1^, 0.002 s^−1^, and 0.02 s^−1^. The RMSE value for the stress by support vector regression was found to be 2.55. The support vector regression could predict the stress with similar accuracy as the neural network model, while being computationally less expensive.

Table 4 shows the RMSEs (root mean square error) for three algorithms.

**Figure 11:**
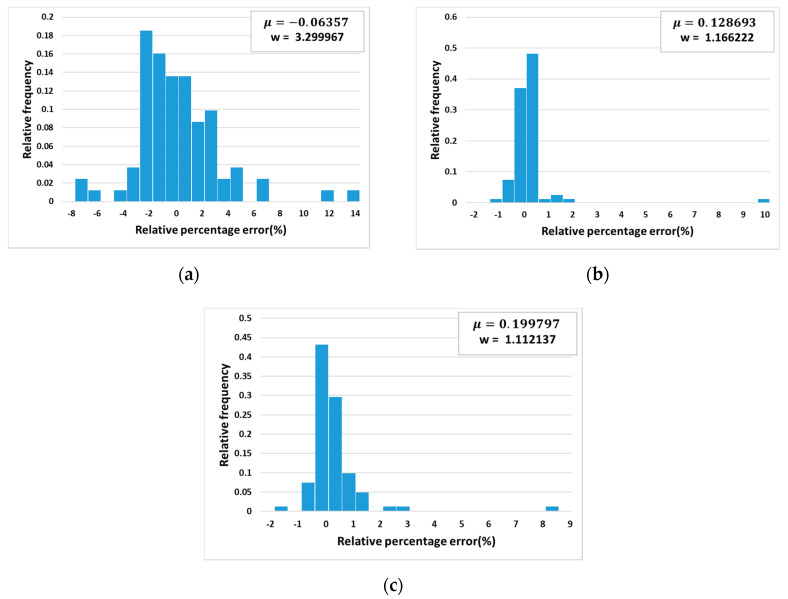
Relative frequency vs. relative percentage error using (**a**) Arrhenius equation; (**b**) Neural network; (**c**) Support vector regression.

In this research, a study on relative percentage errors by three algorithms was done. Figure 11 shows the relative frequency of errors versus relative percentage error using (a) Arrhenius equation, (b) neural networks, and (c) the support vector machine algorithm. The plots (a), (b), and (c) for three algorithms are provided with mean value, and standard deviation of relative percentage errors as Equations (11) and (12):

(11) $$\mu \;=\;\frac{1}{n}\overset{n}{\underset{i=1}{\sum }}\delta_{i}$$

(12) $$w\;=\;\sqrt{\frac{1}{n-1}\overset{n}{\underset{i=1}{\sum }}{\left(\delta_{i}-\mu \right)}^{2}}$$

where μ is the mean value of the relative percentage errors and w is the standard deviation, δ is the relative percentage error, *n* is the number of samples, and *i* is the index of a sample.

The absolute value of the mean relative error by the Arrhenius-type equation was found to be approximately 0.06 which is relatively smaller than the absolute mean values by the neural network approach and support vector machine algorithm. However, the standard deviation was found to be much larger than that of the neural network and support vector machine which means that the errors using the Arrhenius-type equation are more dispersed. The absolute values of mean relative percentage error by the neural network approach and the support vector machine algorithm were found to be slightly larger than that of the Arrhenius equation but the standard deviation value was smaller than that of the Arrhenius equation. Therefore, the performance of the neural network approach and the support vector machine algorithm are considered to be more reliable than the Arrhenius equation, while the support vector machine algorithm has more reliability than the neural network approach.

## 4. Conclusions

This work presents a comparison of three algorithms that can be used for the modeling of the hot deformation flow stress of 316L stainless steel. The Arrhenius equation, neural networks, and support vector regression algorithms are compared in terms of their prediction accuracies and computational efficiencies. The conclusions are presented as follows:

1. The flow stress calculated by the Arrhenius equation was similar to the measured stress. However, the prediction accuracy still needs to be improved.
2. The neural networks could predict the flow stress with better RMSE than the Arrhenius equation. However, the development and the optimization of the neural network model were not efficient. Furthermore, the neural network model was not computationally efficient.
3. The support vector regression algorithm predicted the flow stress with similar RMSE as the neural network model. At the same time, developing the support vector regression model was efficient and computationally inexpensive.
4. The neural networks and support vector regression algorithms predicted the flow stress more reliably than the Arrhenius equation. In addition, the performance of support vector regression was more reliable than the neural network.
5. Although the neural network and the support vector regression algorithms calculated flow stress with better RMSE than the Arrhenius equation, the algorithms could not extrapolate and predict the flow stress outside the range of the training data.

## Figures and Tables

**Table 1:** Parameters obtained for the Arrhenius-type constitutive equation.

Strain	0.05	0.1	0.15	0.2	0.25	0.3	0.35	0.4	0.45
α	0.0091	0.0081	0.0076	0.0073	0.0071	0.007	0.0071	0.0073	0.0081
n	6.0452	5.4551	5.2624	5.1774	5.0818	5.014	4.9624	4.8867	5.2208
Q(kJ)	476.67	446.37	433.83	430.11	426.47	426.65	429.75	432.76	458.30
A	7.3454 × 10^17^	4.737 × 10^16^	1.493 × 10^16^	1.069 × 10^16^	7.41 × 10^15^	7.49 × 10^15^	9.96 × 10^15^	1.21 × 10^16^	9.634 × 10^16^

**Table 2:** Processing parameters for the neural networks.

Parameter	Neurons in Input Layer	Neurons in Hidden Layers	Output Neuron	Epochs	Learning Rate	Weight Decay
Value	3	250,250	1	1500	0.001	0.1

**Table 3:** Parameters for support vector regression.

Parameter	Kernel	C	Epsilon
value	Radial Basis Function	5000	0.003

**Table 4:** Root mean square error using three algorithms.

Arrhenius Equation	Neural Network Model	Support Vector Regression
5.59	2.85	2.55
